# Selection of Neural Oscillatory Features for Human Stress Classification with Single Channel EEG Headset

**DOI:** 10.1155/2018/1049257

**Published:** 2018-12-23

**Authors:** Sanay Muhammad Umar Saeed, Syed Muhammad Anwar, Muhammad Majid, Muhammad Awais, Majdi Alnowami

**Affiliations:** ^1^Department of Computer Engineering, University of Engineering and Technology, Taxila 47050, Pakistan; ^2^Department of Software Engineering, University of Engineering and Technology, Taxila 47050, Pakistan; ^3^Center for Vision, Speech and Signal Processing, University of Surrey, Surrey, GU2 7XH, UK; ^4^Department of Nuclear Engineering, King Abdulaziz University, Jeddah, Saudi Arabia

## Abstract

A study on classification of psychological stress in humans using electroencephalography (EEG) is presented. The stress is classified using a correlation-based feature subset selection method that efficiently reduces the feature vector length. In this study, twenty-eight participants are involved by filling in the perceived stress scale-10 (PSS-10) questionnaire and their EEG is also recorded in closed eye condition to measure the baseline stress. The recorded data is labelled on the basis of the stress level that is indicated by the participant's PSS score. The feature selection method has shown that, among the EEG oscillations, low beta, high beta, and low gamma are the most significant neural oscillations for classifying human stress. The proposed method not only reduces the time to build a classification model but also improves the classification accuracy up to 78.57% using a single channel wearable EEG device.

## 1. Introduction

Rapid changes in technology and society are bringing unavoidable stress to everyday human life. Life itself offers specific emotional and physical challenges. Stress response in humans has been observed by using different psychophysiological systems [1]. When a mental or a physical challenge is presented, a stress response is triggered and several hormones are secreted, including adrenaline, immunoglobulin A (IgA), and cortisol. Consequently, heart rate, pulse, and blood pressure are also increased [2]. Daily life challenges of modern era are causing perceived threats, which constantly arouses the brain. This stress response may not present much harm in the short-term but for longer periods of time that can have some detrimental effects on the health of an individual. Frequent activation of stress response leads towards chronic stress state [3]. The hormones secreted in humans due to stress response for longer periods of time can badly affect the immune system making them vulnerable to infections. Therefore, stress has become a serious issue for human health that can reduce the abilities of an individual, irrespective of their age and gender [4].

Traditionally, stress has been measured using subjective instruments like psychological questionnaires and interview methods, which require major human intervention. Psychological stress tests in connection with electroencephalography (EEG) have been employed in a few researches. In the last decade, quantification of stress using noninvasive physiological sensors has been attracting several researchers [5, 6]. Among other noninvasive techniques, wearable EEG provides good temporal resolution and mobility [7]. An increase in the level of stress can change the underlying EEG oscillations [8]. As the origin of stress response lies in the brain, EEG signal processing becomes a significant technique for the detection and analysis of human stress [2]. It is observed that, under stress condition, the power of alpha band decreases and beta band increases [9]. A higher level of beta waves has been associated with a person in panic condition [10]. Regularity indices like entropies to assess changes in the EEG time series and decreased alertness have also been used to analyse stress [9]. Positive emotions are processed by the left hemisphere and negative emotions are processed by the right hemisphere of brain [11]. The role of prefrontal cortex cannot be negated in the emotional and motivational processes [12]. Therefore, alpha asymmetry based on EEG at the frontal region of brain has been observed to vary statistically under depression [13] and examination stress [14].

Wearable EEG devices have made it possible to monitor stress in daily life activities, as these are unobtrusive, mobile, and usable for a longer duration. Stress and depression related diagnosis suffers from patient denial and biasing in self-reporting; hence EEG based systems have been proposed for a more effective diagnosis [15]. It is believed that for measuring stress reliably, a dense placement of EEG electrodes is required over the human scalp [14]. Clinical EEG systems are dense with 128−, 64−, or 32− channels, which are not easy to wear. Contrary to it, nowadays fewer channel EEG headsets are available commercially. It is reported that a single channel EEG headset can be used at the frontal region for the purpose of emotion recognition [16] and human authentication [17]. Stress response generates neurochemical alteration in the frontal part of the brain [18]. The presence of stress has been considered responsible for an increase in the EEG beta band power [19]. In a closely related study [20], workload and stress states have been differentiated successfully by using a single channel EEG headset.

In this paper, for the first time the importance of low beta, high beta, and low gamma neural oscillations at the frontal site (FP1) is presented as features for classification of perceived human stress. This experiment uses a single channel EEG headset for the purpose of recording brain activity. In this study, an EEG based experiment is performed to collect the data of twenty-eight participants. Perceived stress scale (PSS) questionnaire is a self-report measure of perceived stress, which is used in numerous studies. It has been validated for quantifying levels of chronic stress [21]. The questionnaire is filled by each participant and is used for labelling them as either stressed or nonstressed, based on their PSS score. In closed eye state, baseline EEG signals are recorded to observe the brain activity. These recorded EEG signals are converted into frequency subbands, which are used to create a feature vector. A correlation-based feature subset (CFS) selection method [22] is applied to the feature vector to reduce its length. This results in improved accuracy and efficiency for classification of human stress.

The organization of the paper is as follows. In material and methods section, the proposed stage-wise methodology is presented involving feature extraction, selection, and classification. Experimental results are reported and discussed in results and discussion section, whereas conclusion is presented at the end.

## 2. Materials and Methods

**Figure 1:**
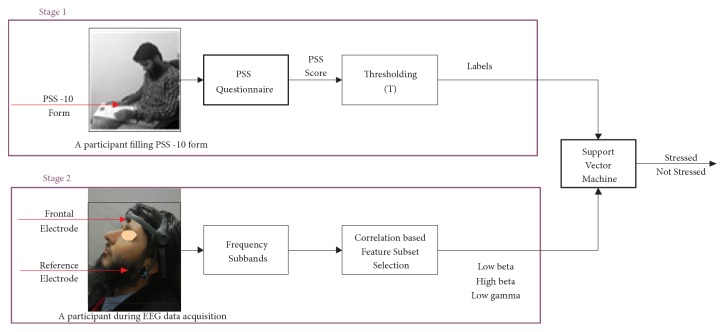
A diagram showing major steps involved for human stress classification using electroencephalography.

The major processing blocks used in this study are shown in Figure 1, which are discussed in detail in the following subsections. An informed consent has been taken from the participant shown in the figure for illustrating the experimental setup of the proposed methodology, where the experimental setup is divided in two stages.

### 2.1. Stage 1

In the stage 1, PSS-10 form was filled by participants involved in the study. The PSS-10 form is comprised of 10 questions. This questionnaire is recommended to be used with participants having a minimum education level of a junior high school. It inquires the participants about experiences during the last month, such as how frequently a stressful event has occurred in this period. The questions are answered on a scale of 0 to 4, where 0 represents no occurrence and 4 represents a very frequent occurrence of a stress related event. Perceived stress level of an individual is represented by the accumulative score of PSS questionnaire. A higher score of PSS means a higher level of perceived stress and vice versa. A total of twenty-eight participants (10 females and 18 males) were involved in the study. Their ages were in the range of 21-34 years and the mean of their ages was 27.5 years. All participants selected for this study belonged to education sector, i.e., either a university student or a faculty member. All participants fulfilled the basic aptness requirement with no reported mental illness. The threshold *T* selected for participants to be in stress was calculated as

(1) $$\begin{array}{c} T=\mu +\frac{\sigma }{2}, \end{array}$$

where *μ* represents the mean and *σ* represents the standard deviation of the PSS-10 scores of all participants. Those participants having a PSS score below *T* are relatively free of stress and those having scores equal or above *T* are comparatively considered to be stressed.

### 2.2. Stage 2

In the stage 2, Neurosky Mindset device, which is a single channel EEG headset, was used to record data of the participants. The headset device provides a single channel of EEG recording from a dry electrode placed at the frontal location, FP1 of the brain, referenced to an electrode placed at the ear lobe, which is shown in Figure 1. The device uses Think-Gear application specific integrated circuit module (TGAM) dry electrode technology that operates at a minimum of 2.7V and covers a bandwidth of 3 − 100Hz. The sampling rate for recording EEG data is configured to 512* Hz*.

The participants were given clear instructions to keep themselves relatively free of thoughts and keep their heads still during the recording session. The easy to wear single channel headset was set up on all participants one by one. EEG signals were recorded for a duration of three minutes in closed eye condition. All EEG recordings were performed in lab settings, where lighting conditions were kept similar. A peaceful environment was provided to avoid artefacts in the EEG recordings. The experimental procedure is approved by the board of postgraduate studies at the University of Engineering and Technology, Taxila, and follows the Helsinki declaration.

### 2.3. Feature Extraction and Selection

Features are extracted by applying fast Fourier transform (FFT) over the recorded EEG signal, to transform this data in frequency domain. Moreover, the feature vector based on the neural oscillations is created by applying band-pass filters to extract the frequency subbands. The oscillatory subbands for the EEG signals and their frequency ranges are shown in Table 1. A third-party software designed to work with the Neurosky headset, Myndplay Pro (available at https://store.neurosky.com/products/myndplay-pro), was used for extracting these frequency subbands. This software records EEG data and provides output for EEG frequency subbands in CSV format. The power in each of these frequency subbands is normalized in the range of 0 to 1 by using

(2) $$\begin{array}{c} n_{p}=\frac{x-x_{min}}{x_{max}-x_{min}}, \end{array}$$

where *n*_*p*_ is the normalized power,* x *is an instance of power, *x*_*min*_ is the minimum power, and *x*_*max*_ is the maximum power value of all instances.

The correlation-based feature subset selection method uses feature to feature and feature to class correlation for identifying significant features [22]. It is a widely used method in machine learning for feature selection and reduction. This feature selection technique is for discrete-class supervised learning. The technique is based on the assumption that correlation of useful feature subsets is low with each other but feature members of these subsets are predictive of class. The CFS method is based on a test theory that computes merit of the feature subset heuristically from pair-wise feature correlations in a reasonable time. After performing heuristic search, it reports the subset with the highest merit. The merit of subset *E* containing *k* features is calculated as

(3) $$\begin{array}{c} {Merit}_{E_{k}}=\frac{kr_{c}f}{\sqrt{k+k\left(k-1\right)r_{f}f}}, \end{array}$$

where *Merit*_*E*_*k*__ represents merit of a feature subset *E*, which consists of *k* features, *r*_*c*_*f* is the average of correlation of features with respect to class *f* ∈ *E*, and *r*_*f*_*f* is the average of correlation of features with respect to features. The numerator of (3) indicates the power of a set of features to predict a class, whereas the denominator represents the redundancy among the features. This method is applied over the oscillatory subbands and resulted in the selection of low beta, high beta, and low gamma as significant neural oscillations for the classification of human stress.

### 2.4. Classification

Support vector machine (SVM) is used for the stress classification task in this study, since it has been used successfully in previous studies [22]. SVM is an algorithm that involves supervised learning for classification and regression task and has been used in the development of models that analyse baseline human stress. It works by determining a linearly separating hyper-plane in a higher dimension by making use of support vectors. This hyperplane separates two classes of data if the training data is transformed satisfactorily to a higher dimension. In this study, the sequential minimal optimization algorithm with a sigmoid kernel is employed. The scoring function for SVM is given as,

(4) $$\begin{array}{c} y=\overset{n}{\underset{1}{\sum }}a_{i}y^{\left(i\right)}K\left(\;f^{i},f\;\right)b, \end{array}$$

where *y*^(*i*)^ represents a label of class and *f*^(*i*)^ is an input feature, *K* is the kernel function, *a*_*i*_ is the coefficient associated with input vector, and *b* is an arbitrary scalar value. If the scoring function, *y*, is negative it belongs to one class and if it is positive it belongs to the other class. In our case, one class is stressed and the other class is nonstressed.

## 3. Results and Discussion

The evaluation parameters, experimental results, and comparison of the results with other reported methods are presented in the following subsections.

### 3.1. Evaluation Parameters

The performance of the system is tested using various performance measures. The accuracy, ACC, is determined as

(5) $$\begin{array}{c} ACC=\frac{TP+TN}{TP+FN+FP+TN}, \end{array}$$

where TP and TN represent true positive and true negative values,respectively, and FP and FN denote false positive and false negative values, respectively. The kappa coefficient, *Ƙ*, is independent of the number of samples per class and the number of classes, where *Ƙ* = 0 means chance level performance and *Ƙ* =1 means perfect classification. A value of *Ƙ* < 0 means that classification performance is worse than chance. If ACC is the accuracy and ACC_0_ is the reciprocal of the number of classes, Kappa coefficient is calculated as

(6) $$\begin{array}{c} Ƙ=\frac{ACC-{ACC}_{0}}{1-{ACC}_{0}} \end{array}$$

F-measure is generally considered as a harmonic mean of precision and recall of a system. The range of its value is from 0 to 1. A higher value means better precision and recall of a system. F-measure is calculated by using

(7) $$\begin{array}{c} F-Measure=2\times \frac{Precision\times Recall}{Precision+Recall}, \end{array}$$

where Precision equals *TP*/(*TP* + *FP*) and recall equals *TP*/(*TP* + *FN*). The root absolute error (RAE) and root mean squared error (RMSE) are computed as

(8) RAE=∑inoi−ai∑inO−i−ai,

(9) RMSE=∑i=1noi−ai2n,

where o_i_ is the observed outcome, ${\overset{-}{O}}_{i}$ is the average of outcomes, *a*_*i*_ is the actual outcome, and *n* is the total number of observations.

### 3.2. Data Labelling and Processing

**Figure 2:**
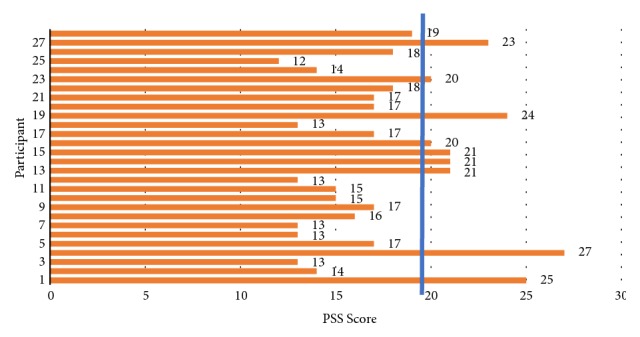
PSS scores for the participants involved in the study. The horizontal line represents the threshold value.

In this section, the results of CFS selection algorithm are presented. Based on these features, the performance parameters are compared with related studies using single channel headset. Trends of different neural oscillations are observed at the end of this section to validate the results of conducted study with respect to the studies performed with a larger number of electrodes. The analysis of responses recorded from PSS-10 questionnaire indicated a mean (*μ*) value of 17.6 and a standard deviation (*σ*) of 4.04, respectively. The threshold *T* for grouping of participants into stressed and not stressed groups is calculated as 19.6. The PSS scores are shown in Figure 2. Those participants with a PSS score above the threshold value are categorized as stressed, whereas the rest are labelled as nonstressed.

**Figure 3:**
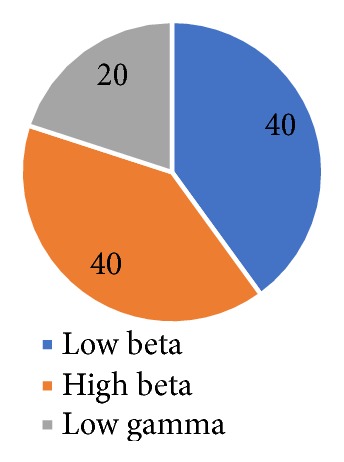
A breakdown in terms of the percentage of selected EEG subband oscillatory features by using correlation-based feature subset selection.

The results of applying CFS over neural oscillations are shown in Figure 3 in terms of percentage of selected features from each frequency subband. It is evident that 40% of the selected features belong to high beta oscillation, 40% belong to low gamma oscillation, and 20% are selected from low beta oscillation, whereas, no features have been selected by CFS from any other neural oscillations. Based on these results, low beta, high beta, and low gamma oscillations are selected as features for the classification of human stress. To validate the classification results, a 10-fold cross validation is used. In this technique, the input data is equally partitioned into 10 parts, 9 parts of data are used for the training and remaining 1 part of data is used for the testing. This process is performed repeatedly by using all combinations of test and training data, and finally averaged results are reported. All experiments conducted in this study are implemented in Waikato environment for knowledge analysis, version 3.8.1, which is a popular platform for machine learning and is written in JAVA language [26]. The SVM classifier is used using the polynomial kernel with a cache size, C=250007 and calibrator settings as logistic, using number of decimal places equal to 4.

## 4. Discussion

Table 2 compares the performance of the algorithms used in single channel-based studies for classification of human stress in terms of various performance measures. The execution times for [23, 24] and the proposed methodology are computed on an Intel i5 system with RAM of 4 GB. It is observed that the SVM algorithm, when used with the proposed features selected by CFS, gives 7.1% higher accuracy as compared to the case, where all neural oscillations are used as features. Moreover, the Kappa statistic is improved to 0.44 from 0.20, which indicates that the classification accuracy is now much better than chance. The F-measure in this case is improved to 0.662 from 0.53, which clearly indicates an improvement in the precision and recall of stress classification system. The values of RMSE and RAE are also reduced as a result. Furthermore, the classification time is reduced by a factor of 8 as compared to [23], since the number of significant oscillatory features is reduced from 8 to 3 by using CFS, namely, low beta, high beta, and low gamma oscillations.

Similarly, classification accuracy is improved by 7.1% as compared to the study [24], in which low beta is used as a sole feature for classification. F-measure of proposed features is equal to that of low beta-based classification. RMSE is lowered to 48.46 from 52. However, the number of features used is increased from one to three and so is the time to build classification model. Classification accuracy is improved by 6.5% in proposed methodology as compared to [25], in which discrete cosine transform of EEG signal has been used. Our proposed method suggests SVM for classification, while in [25], KNN is used for classification. However, the accuracy is 83.33% for workload and stress classification using the Naive Bayes classifier with EEG based features [20]. Intrinsic mode functions are used as features in addition to delta, theta, alpha, and beta subbands with stress and workload inducing task. The reported accuracy for workload classification using only EEG features is 86.66%. Comparatively, baseline EEG is used for the classification of perceived stress in closed-eye condition in this study, which does not rely on stress inducing tasks.

**Figure 4:**
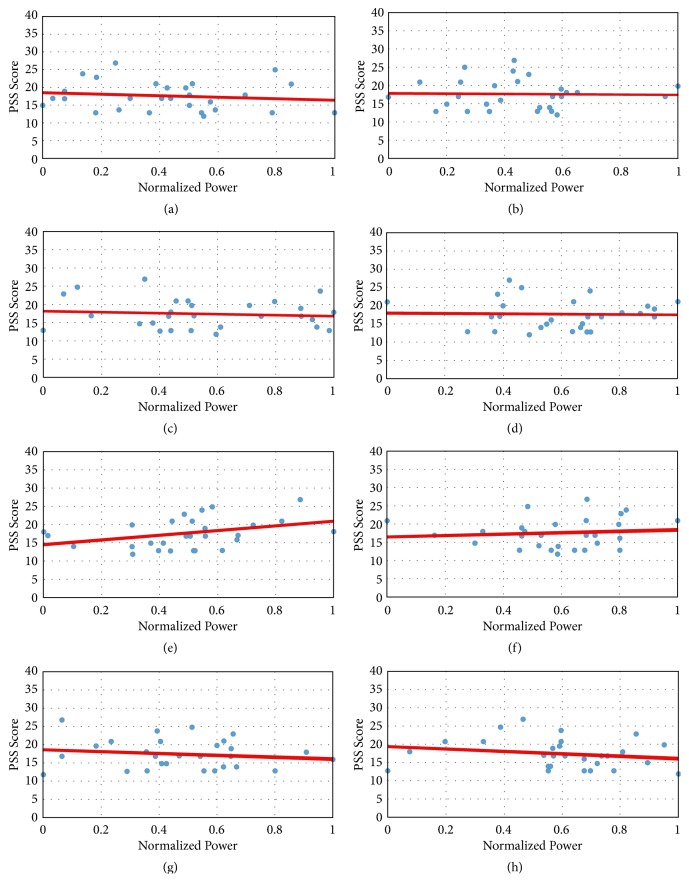
Trend lines showing the normalized power and the PSS scores for all participants in oscillatory band (a) delta, (b) theta, (c) low alpha, (d) high alph,a (e) low beta, (f) high beta, (g) low gamma, and (h) mid gamma.

The relationship between normalized power of each frequency subband and PSS score is shown in Figure 4. An average value of the normalized power for each frequency subband is calculated for each participant. These power values are scattered such that the x-axis represents the normalized power value and the y-axis represents the PSS score. The trends in these powers and the PSS values are observed by drawing trend lines on the scattered data. A general trend of decrease in normalized power is observed with the increase in PSS score, when all oscillations are considered except beta oscillations. This trend can be easily observed in Figures 4(a), 4(b), 4(c), 4(d), 4(g) and 4(h) for delta, theta, low alpha, high alpha, low gamma, and mid-gamma oscillations, respectively. The trend lines for the beta oscillations, Figures 4(e) and 4(f) show an increase, conforming to the fact that an increase in stress shows an increase in the power of beta band as described in [9].

The variation in the powers of delta, theta, low alpha, and high alpha waves with respect to PSS score is not considerable. The trend of gamma power shows higher values for lower PSS score. Relative gamma in the prefrontal region is shown to correlate with induced stress [27], although this EEG marker requires inducing stress in the subject. The proposed method measures baseline stress without inducing any stress using a stressor. Therefore, it is more suited for day to day activities in which there is not a stressor involved. These trends using single electrode device show a similar trend as shown by studies performed with multiple electrodes [8, 9]. Hence, a low channel wearable device like single channel Neurosky Mindwave headset can be used for human stress analysis system. Such a headset is easy to wear as it uses a dry electrode for recording of EEG signals.

## 5. Conclusions

In this paper, an EEG based human stress classification system is proposed that uses neural oscillation as features. The recorded EEG data of participants are labelled into two classes, i.e., stressed and nonstressed, using their perceived stress score. It has been shown that, for a single channel EEG headset, CFS reduced feature vector from eight to three neural oscillations, comprising low beta, high beta, and low gamma for effective classification of human stress. It not only reduced the computational time by a factor of 8 but also improved the accuracy of the SVM classifier by 7.1%. The results have shown significant values for various performance measures used to test the stress classification system. The reduction in feature set also leads to reduction in computational cost. This fact leads to potential real time application in both monitoring and identification of stress in individuals using easy to wear commercially available EEG headset. A complete system that can use such arrangements to monitor and manage daily life stress requires being more versatile in terms of the long-term wearability of EEG device and this is a challenge that needs to be addressed.

## Figures and Tables

**Table 1:** Frequency bands of the EEG oscillatory subbands involved in feature vector creation to classify stress.

Sr. No.	Neural Oscillations	Bands
1	Delta	1*Hz*− 3*Hz*
2	Theta	4*Hz*− 7*Hz*
3	Low Alpha	8*Hz*− 9*Hz*
4	High Alpha	10*Hz*− 12*Hz*
5	Low beta	13*Hz*− 17*Hz*
6	High beta	18*Hz*− 30*Hz*
7	Low Gamma	31*Hz*− 40*Hz*
8	Mid Gamma	41*Hz*− 50*Hz*

**Table 2:** A comparison of the performance parameters of algorithm used for classification of human stress using single channel headset.

Neural Oscillations	ACC (%)	Kappa	F-Measure	Time (sec)	RMSE	RAE	Classifier
All Oscillations ([23])	71.43	0.21	0.531	0.080	64.61	114.03	Naive Bayes
Low Beta ([24])	71.43	0.2	0.662	0.004	52.00	65.29	SVM
Discrete Cosine Transform ([25])	72.00	−	−	−	−	−	K-nearest Neighbour
delta, theta, alpha and beta and IMFs ([20])	83.33	−	−	−	−	−	Naive Bayes
**Low Beta, High Beta, Low Gamma (Proposed)**	**78.57**	**0.44**	**0.662**	**0.010**	**48.46**	**98.75**	**SVM**

## Data Availability

The data used in this study will be made available online on acceptance.
